# Iatrogenic Displacement of a Foreign Body into the Periapical Tissues

**DOI:** 10.1155/2014/698538

**Published:** 2014-11-11

**Authors:** Hugo Plascencia, Alvaro Cruz, Rodrigo Solís, Mariana Díaz, Josué Vázquez

**Affiliations:** ^1^Endodontic Postgraduate Program, CUCS, University of Guadalajara, Guadalajara, JAL 44340, Mexico; ^2^Biomedical Sciences Research Institute, Endodontic Postgraduate Program, CUCS, University of Guadalajara, Guadalajara, JAL 44340, Mexico

## Abstract

The presence of a foreign body in the periapical tissues can cause endodontic failure by triggering an inflammatory response and a subsequent foreign body reaction. This inflammatory response, which can occur to varying degrees, appears radiographically as a radiolucency that can remain asymptomatic for many years. A foreign object can reach the apical region by accident or iatrogenic procedures during dental procedures. The aim of the present case report is to describe the endodontic surgical treatment of an iatrogenic displacement of a foreign body (a metal fragment) into the periapical tissues and to describe its clinical and radiographic follow-up over a period of 52 months.

## 1. Introduction

Root canal therapy is a reliable and predictable procedure with high short- and long-term success rates; nevertheless, endodontic failure can occasionally result from this treatment. Endodontic failure is usually associated with the persistence of bacteria within the root canal system [1]; however, Nair [2] has reported that another less common cause of endodontic failure is the presence of a foreign body in the periapical tissues.

The apical periodontium can encapsulate different irritants with collagen fibers and even has been shown to heal in the presence of a foreign object [3]. Nonetheless, an intense inflammatory response characterized by abundance of macrophages and giant cells and a subsequent foreign body reaction can also be observed. This inflammatory response is often associated with the formation of a completely asymptomatic periapical radiolucent lesion [4, 5].

Foreign bodies can be forced into the periapical region by three different ways: (i) compacting food in teeth with a necrotic pulp and destroying the pulp chamber [6], (ii) a patient intentionally introducing objects into his or her mouth [7], and (iii) accidentally displacing a foreign body during dental procedures [8]. Therefore, the aim of this case report is to describe the endodontic surgical treatment and subsequent long-term clinical and radiographic follow-up of an iatrogenic displacement of a foreign body into the periapical tissues through the root canal.

## 2. Case Presentation

Our case is of a 25-year-old male patient who came into the clinic for a dental check-up of tooth number 21 (maxillary left central incisor), which presented a gradual colour change of the clinical crown with regard to the neighbor teeth over the past three years. Although this tooth was completely asymptomatic, the patient reported that he had suffered dental trauma 15 years ago when he fell while practicing sports, resulting in a complicated crown fracture. The day after the accident, his parents took him to see a dentist who simply “placed a screw and sealed the tooth with tooth-colored material.” The medical history revealed no history of systemic problems or allergies.

**Figure 1:**
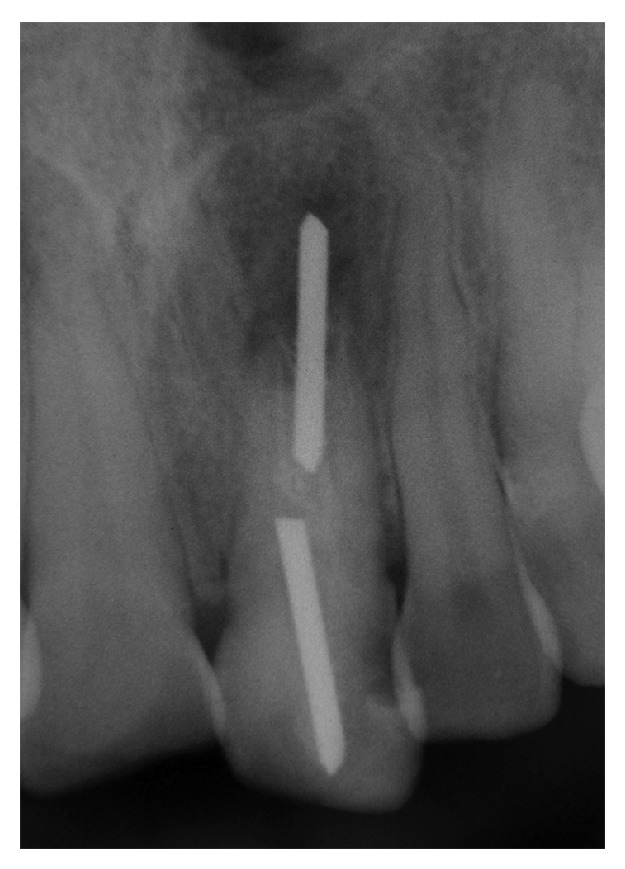
The initial radiograph showing the radiopaque body invading the periapical tissues.

Upon clinical inspection, a resin was observed at the palatal area and incisal edge, and the buccal and palatal mucosa appeared normal. There was no response to vertical or horizontal percussion or to palpation of the mucosa. Probing depth and tooth mobility were both within normal limits, and the adjacent teeth responded normally to both thermal (Endo-Ice, Hygenic, USA) and electrical (Digitest, Parkell, USA) tests. The radiographic inspection revealed the presence of an intraradicular post at the crown level, which was surrounded until the middle third of the root by sealing material (Figure 1). A wide canal and an immature apex were detected. In addition, a radiopaque foreign object (approximately 10 mm in length and 1.5 mm in width) was also detected in the apical region of the immature canal. This foreign object was also displaced approximately 5 mm into the periapical tissues and was surrounded by a diffuse radiolucent periapical lesion. The morphology of the foreign object was very similar to that of the intraradicular post identified at the crown level. In view of this finding and based on the history reported by the patient, we assumed that his previous dentist had not been fully aware of the association between the patient's age and the immature apex while performing dental treatment 15 years earlier and accidentally forced the foreign object that is intended to be used as intraradicular post through the apex and into the periapical tissues while attempting to remove it. Consequently, the dentist likely inserted another intraradicular post and sealed the access cavity with resin.

Once all of the aforementioned information was collected, a pulpal diagnosis of previously initiated endodontic therapy with signs of infection was established, with periapical diagnosis of chronic apical periodontitis combined with iatrogenic displacement of a foreign body into the periapical tissues. Thereby, we decided to perform a surgical endodontic treatment of the tooth, including the retrograde removal of the foreign object, retrograde instrumentation, and retrograde obturation with a thermoplasticized gutta-percha injection. We concluded that trying to remove the foreign object using an orthograde approach could weaken the fragile immature root structure and could risk sending the object into a deeper anatomic area, such as the floor of the nasal cavity. The intentional replantation was considered only a last option due to the high probability of root fracture at the time of extraction. The initial prognosis for this patient was favourable.

### 2.1. Endodontic Surgical Treatment

**Figure 2:**
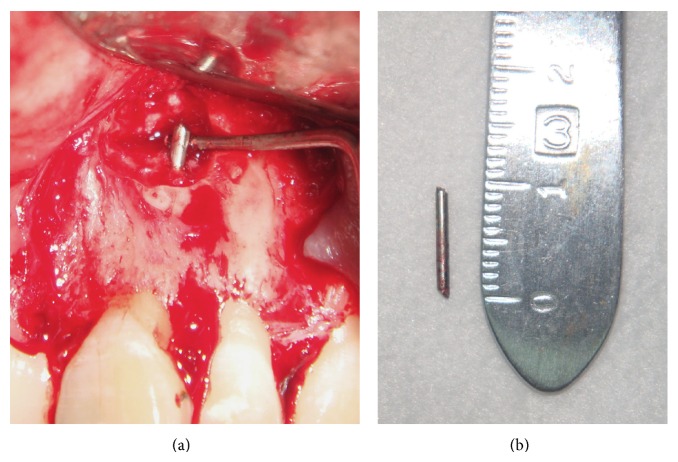
(a) The clinical appearance of the metal fragment located in the periapical tissues. (b) An image showing the length (approximately 10 mm) of the metal foreign body that was removed during the surgical procedure.

**Figure 3:**
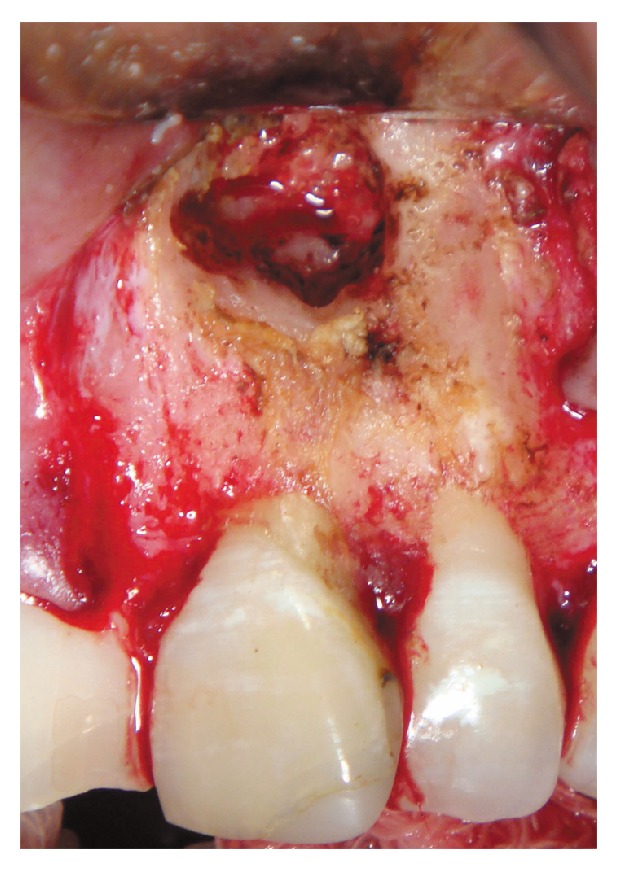
Clinical appearance of the surgical socket after the removal of the metal fragment.

After performing a regional anaesthetic block with 1 : 100,000 articaine and waiting 10 min for deep action, a total thickness triangular flap was raised and the root apex was located with the use of long-shank round carbide bur number 4 mounted on a high speed hand piece with no direct air to the working area, under abundant saline irrigation delivered with a hypodermic syringe. After locating and exposing the periapical lesion, all tissue within the bony crypt was removed with a curette to locate the foreign body (metal fragment) (Figure 2(a)), which was easily removed with a hemostat (Figure 2(b)). Because the tooth had an incomplete root formation, once the bleeding was under control with the use of gauze impregnated with ferric sulphate, the root apex was remodeled only slightly with a Zekrya bur at high speed (Figure 3). Due to the fact that there was still no apex and the apical deltas were not yet fully formed, the apical reduction of the last 3 mm was avoided.

**Figure 4:**
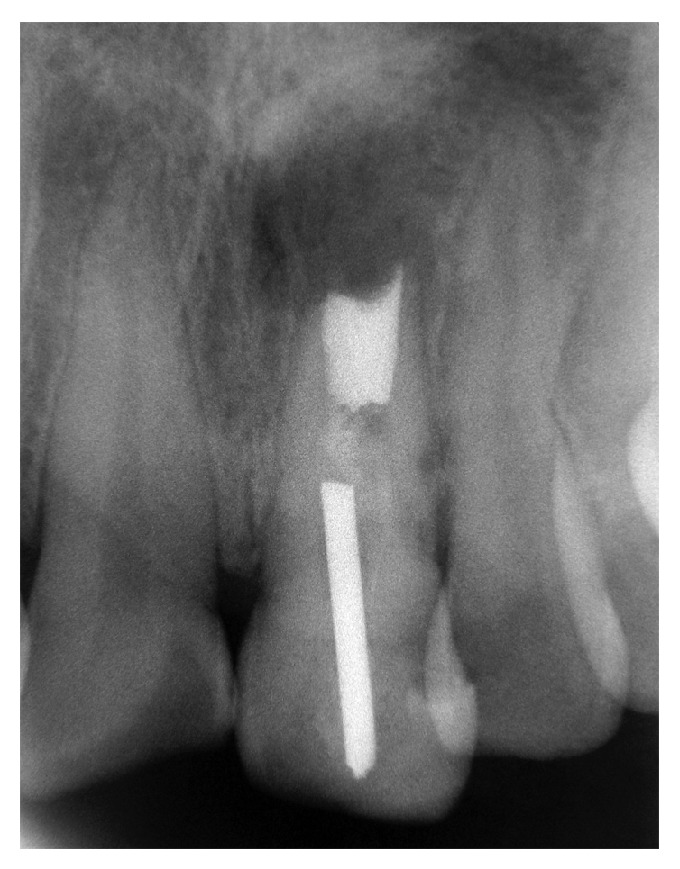
A postoperative radiograph shows the proper retrograde placement of thermoplasticized gutta-percha in direct contact with the resin located inside the main canal.

Resin was detected inside the root canal using a retrograde approach with an endodontic explorer; thus, there was no need for a retrograde preparation of the entire length of the main canal. The ultrasonic retrograde preparation of the 3 mm apical portion was performed using ultrasonic tips (Endo retrograde Kit, NSK, Brasseler USA). Low intensity and very light pressure were used to prevent the creation of microfractures in the thin walls of the immature apex, and abundant saline irrigation was provided drop by drop at a distance. After drying the retrograde cavity with absorbent paper points, the cavity was filled by retrograde obturation with thermoplasticized injected gutta-percha (Obtura System II, SybronEndo, Coppell, Texas, USA), which was compacted and its placement was confirmed radiographically once it had cooled (Figure 4). The flap was approximated and sutured gently with 5-0 nylon separate stitches. Following the procedure, the patient was provided with oral and written postoperative instructions and drug treatment. The patient returned after three days for suture removal and did not report any unusual discomfort. The histopathological diagnosis of the lesion was periapical granuloma.

### 2.2. Clinical and Radiographic Follow-Up

**Figure 5:**
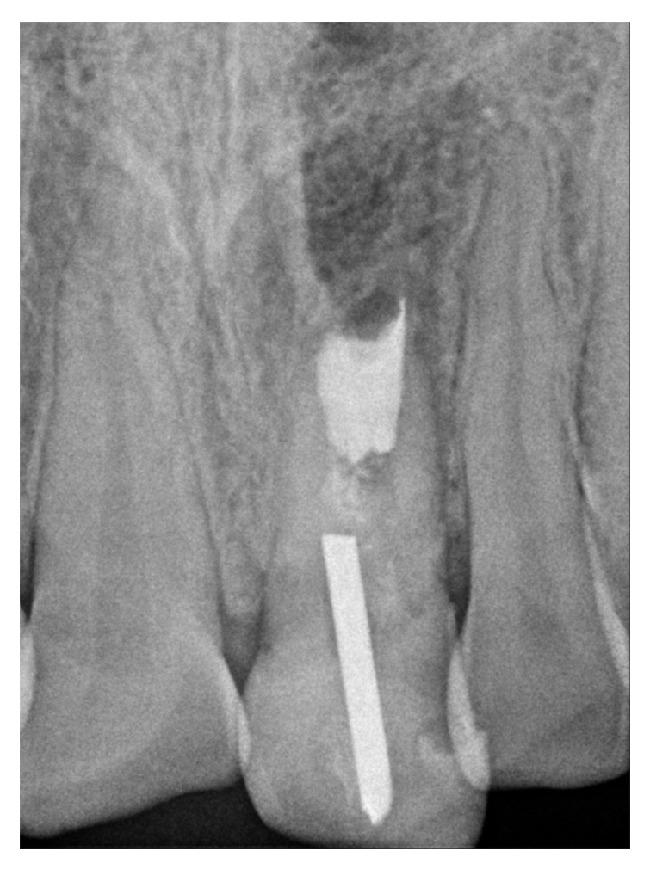
A follow-up radiograph at 12 months reveals a marked decrease of the periapical lesion, which was in the process of healing.

**Figure 6:**
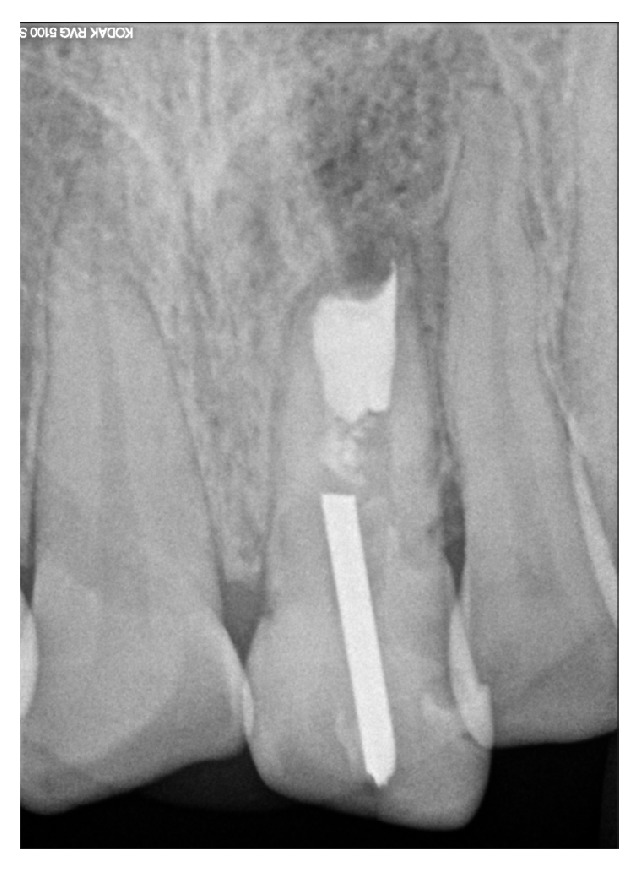
A follow-up radiograph at 52 months (4 years and 4 months) reveals complete healing of the initial periapical bone defect. At this time, the case was considered to be healed.

One month later, the patient returned completely free of any symptoms and signs of infection, and probing depth and tooth mobility were both within normal limits. It was decided to change the crown restoration to prevent the coronal leakage of saliva; however, the patient returned for a follow-up after 12 months, at which time proper radiographic healing of the periapical lesion was observed, and no symptoms or signs of infection were found (Figure 5) and clinically there was no clinical coronal leakage. The patient was advised of the need to change the crown restoration and was asked to return for a follow-up after completing the reconstruction. Unfortunately, the patient ignored the previous recommendation and returned for a follow-up 52 months later. In spite of this, no clinical symptoms or signs of infection were observed. Probing depth and tooth mobility were again within normal limits, and radiographs revealed adequate periapical healing (Figure 6). Therefore, based on the extensive follow-up period and the positive clinical and radiographic features, we considered the patient healed.

## 3. Discussion

The expulsion of a foreign body to the periapical region is an unfortunate accident that can occur during dental procedures and could cause endodontic failure by triggering inflammation and a foreign body reaction [4, 5].

In this case, conservative treatment using an orthograde approach implies high risks, due to the fact that an incomplete root formation is present, because the object can move into deeper anatomical areas, cause excessive wear of the main root canal, or even fracture the fragile structure of the immature root, despite the use of a dental operating microscope. Fortunately, these types of accidents are rare, and relatively few have been reported in the literature [7, 9, 10]. However, the authors describing these cases advise conducting apical surgery first and leaving intentional replantation as a second-line measure.

For many years, the appropriate selection of retrograde filling material has been the subject of debate. The use of gutta-percha for this purpose was considered a good choice because it is biocompatible with human tissues, can be adapted to the irregular walls of the retrograde cavity, is malleable, can absorb some moisture of the periapical tissues, and does not corrode [11, 12]. Goldberg et al. [13] and Witherspoon and Gutmann [14] reported an acceptable tolerance of periapical tissues when placed in direct contact with the gutta-percha used as retrograde filling material in dogs. Both studies reported good results of bone apposition, reformation of the periodontal ligament, and deposition of new cementum on the sectioned root-end. However, a fibrous capsule with mild chronic inflammation was also constantly detected adjacent to the retrograde filling material.

A well-established alternative, mineral trioxide aggregate (MTA), provides good clinical and histological results, which makes it the retrograde filling material of choice, largely due to its physical/chemical/biological properties [15]. However, Tsesis et al. [16] conducted a recent systematic review that evaluated several previous literature reviews and meta-analyses about the prognosis of this condition with modern endodontic surgery. Among the many results obtained, they reported a clinical and radiographic success rate that was very similar after 1 year regardless of whether the surgery was performed with gutta-percha or MTA as the retrograde filling material (88.5% and 90.8% success rate, resp.). However, it is important to note that the use of gutta-percha should be considered only a second-line alternative to MTA in such cases.

This case shows that the iatrogenic displacement of a foreign body into the periapical tissues through an immature root canal can be treated successfully through surgical endodontic treatment, as shown by the clinical and radiographic follow-up after 52 months.
